# Editorial: Application and innovation of multiomics technologies in clinical oncology

**DOI:** 10.3389/fonc.2023.1179829

**Published:** 2023-03-28

**Authors:** Xinmin Li, Anton Budzin, Ye Wang

**Affiliations:** ^1^ Department of Pathology & Laboratory Medicine, Technology Center for Genomics & Bioinformatics, University of California, Los Angeles (UCLA), Los Angeles, CA, United States; ^2^ Laboratory for Translational Genomic Bioinformatics, Moscow Institute of Physics and Technology, Dolgoprudny, Moscow Region, Russia; ^3^ World-Class Research Center, I.M. Sechenov First Moscow State Medical University, Moscow, Russia; ^4^ World-Class Research Center “Digital Biodesign And Personalized Healthcare”, Sechenov First Moscow State Medical University, Moscow, Russia; ^5^ Clinical Laboratory, Qingdao Central Hospital, The Second Affiliated Hospital of Medical College of Qingdao University, Qingdao, China

**Keywords:** clinical oncology, multiomics technologies, genomics, transcriptomics, biomarkers

Cancer is a complex disease that involves multiple layers of genetic, epigenetic, and metabolic alterations and interactions to orchestrate tumor growth and progression. Multiomics technologies empower researchers to generate and integrate these different types of data to provide coherent and complete picture of cancer biology, and to effectively identify key molecular, genetic alterations, pathways, and networks that drive cancer development and progression. These findings can lead to the discovery of new biomarkers, new diagnostic tools, new strategies to overcome resistance and treat cancer patients.

This special issue entitled “Application and Innovation of Multiomics Technologies in Clinical Oncology” compiled 43 articles, highlighting different applications and findings using genomics, transcriptomics and multiomics in a wide range of cancer types. One of the powerful and common omics’applications is its ability to identify molecular signatures and biomarkers for cancer classification, metastasis, prognosis and treatment. This special issue provides ample examples of such, including miR-139-5p and a 3-miRNA signature as potential biomarkers for subtyping breast carcinoma (Yan et al.) and predicting survival in nasopharyngeal carcinoma (Zhou et al.), LncRNA-AC02278.4 and ASF1B as Prognostic Biomarkers for Tumor Growth and Metastasis in Lung Adenocarcinoma (Chen et al.), and poor outcome in the HBV-infected hepatocellular carcinoma (Wang et al.), immune-related and energy metablolism-related gene signatures with prognostic potential for pancreatic cancer (Chen et al.) and hepatocellular carcinoma (Liu et al.).

Of particular interest, Huang et al. used univariable Cox regression analyses and differential gene expression analysis with bulk RNAseq data to identify eight genes that are closely associated with the recurrence-free survivals (RFS) of HCC patients in both training and three validation cohorts. They subsequently built a multivariable Cox model based on the expression of these genes to predict the risk of HCC recurrence. The results showed that the Eight-Gene Cox (EGC) model outperformed other published models and could serve as independent predictor in predicting HCC recurrence. Furthermore, the authors investigated the cell-type-specific expression patterns and their biological functions of these eight marker genes in tumor microenvironment using single-cell RNA sequencing data. Interestingly, these genes had different expression patterns in different cell types. For example, PLCB1 and SLC22A7 were predominantly expressed in malignant cells while FASLG and IL2RB were specifically expressed in T cells, suggesting their functional roles in these given cell types.

The results of this study are not only significant in terms of providing a novel model for HCC recurrence prediction but also in shedding light on the underlying mechanisms behind HCC recurrence. It demonstrates the importance of the tumor microenvironment and highlights the need for a more comprehensive understanding of the interplay between tumor cells and their surrounding microenvironment in cancer development and progression. The insights gained into the functional roles of the HCC recurrence marker genes provide clues for further research and the development of new therapeutic strategies for the treatment of HCC.

In addition to gene expression signatures and biomarkers, the DNA markers are equally informative for predicting treatment efficacy, toxicity and clinical outcome. He et al. identified genetic determinants that can explain individual variations in radio-sensitivity and radiotherapy-associated toxicity in 122 patients with unresectable stage III non-small-cell lung cancer (NSCLC). The research confirmed previously reported genetic association with clinical outcomes and also identified the *FGFR* family genes, *MET*, *PTEN*, and *NOTCH2* as potential novel and independent risk factors of poor post-CRT survival by using a 474-cancer and radiotherapy-related gene panel. These novel biomarkers may offer new therapeutic targets for patients with NSCLC. The identification of genetic markers in relation to radiotherapy-associated thoracic toxicity is critical for the design of better treatment strategies to reduce or eliminate the risk of toxicities associated with CRT.

This study has several important clinical implications. It demonstrated the clinical utility of focused NGS panels in identifying predictive biomarkers for response to CRT, reinforced the importance of pre-treatment genetic information to better inform CRT outcomes and clinical actions in stage III unresectable NSCLCs, and stressed that integrated analysis of multiple alterations can lead to improved stratification of the risk populations.

The utilization of a multi-omics approach has also facilitated the comprehension of molecular pathways, mechanisms, and mechanistic models for tumor development by compiling various types of data and constructing new data-driven concepts. In this Research Topic, such efforts were documented and published for several common and rare cancers, including colon (Wang et al.), gastric (Gao and Yang, 2022), bladder (Mao et al.), nasopharyngeal carcinoma (Lu et al.), and adult spinal intramedullary astrocytomas (Konovalov et al.), the latter paper is supplemented with the first-of-its-kind collection of experimental RNAseq profiles for this orphan cancer.

As the field of omics continues to evolve and expand, a multiomics approach is becoming increasingly important for advancing our understanding of complex diseases such as cancer. By embracing a multiomics approach that combine and integrate multiple layers of molecular information, we can accelerate the pace of discovery and translation of omics research into clinical practice in cancer.

## Author contributions

XL, AB, and YW: writing original draft and editing. All authors contributed to the article and approved the submitted version.

